# Realtime MR guided endomyocardial biopsy with an active visualization bioptome

**DOI:** 10.1186/1532-429X-17-S1-P235

**Published:** 2015-02-03

**Authors:** Toby Rogers, Parag Karmarkar, Ozgur Kocaturk, Kanishka Ratnayaka, Michael Hansen, Anthony Z Faranesh, Robert J Lederman

**Affiliations:** National Heart Lung and Blood Institute, National Institues of Health, Bethesda, MD USA; MRI Interventions, Memphis, TN USA; Institute of Biomedical Engineering, Bogazici University, Istanbul, Turkey; Department of Cardiology, Children’s National Medical Center, Washington, DC USA

## Background

Invasive endomyocardial biopsy is an important tool to diagnose cardiomyopathy. But the diagnostic yield is low because the procedure is performed ‘blind' using, particularly in processes characterized by heterogeneous myocardial involvement. MRI tissue characterization techniques, using late gadolinium enhancement or T1 mapping for example, can identify affected regions and guide biopsy. We performed realtime MR guided transcatheter endomyocardial biopsy using a novel active visualization MR conditional bioptome in swine.

## Methods

An active visualization MR conditional bioptome was designed and built for transcatheter endomyocardial biopsy. All materials were chosen to minimize MR imaging artifacts and to minimize device heating during use in the MR environment. Active visualization of the bioptome shaft was achieved by embedding a loopless dipole RF antenna. The bioptome was tested in a phantom and in vivo in swine to perform transcatheter right and left ventricular endomyocardial biopsy under realtime MR guidance.

## Results

**Figure 1:**
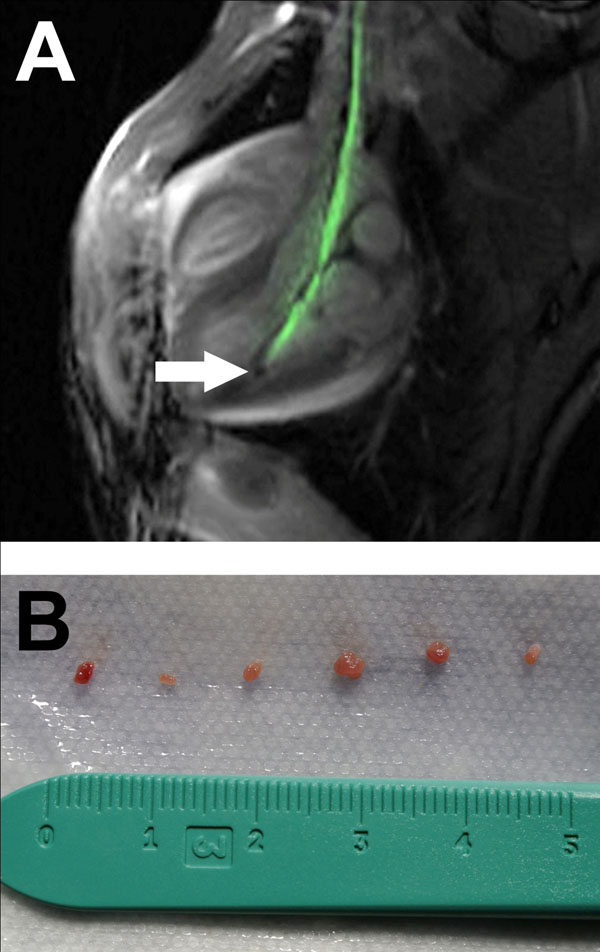
Figure 1

The bioptome shaft was actively visualized under realtime MR imaging. The jaws appeared as a distinct signal void in the phantom and in vivo (arrow, Figure 1A). Device heating was negligible in a phantom. The bioptome was navigated to the right ventricular outflow tract and left ventricle through a standard 7Fr multipurpose curve biopsy sheath. Using the curve of the sheath, it was possible to direct the bioptome to different locations with the ventricles and perform biopsy. Multiple biopsy samples were obtained to demonstrate cutting ability of the bioptome jaws (Figure 1B). Future iterations of the device will include a deflection mechanism and additional active visualization of the jaws to confirm open/closed position.

## Conclusions

We have developed and tested in vivo an active visualization MR conditional endomyocardial bioptome. We have demonstrated clear visualization of the bioptome shaft and jaws under realtime MR guidance. In an animal experiment, the bioptome was steered to different locations with the ventricles and multiple biopsy samples were obtained.

## Funding

This work was supported by the Division of Intramural Research, National Heart Lung and Blood Institute, National Institutes of Health (Z01-HL005062) and by a SBIR contract to MRI Interventions.

